# Fin-Ray soft gripper for object manipulation with multi-robot systems

**DOI:** 10.1016/j.ohx.2026.e00806

**Published:** 2026-07-11

**Authors:** Santiago Velasquez, Alejandro Toro-Ossaba, Daniel Sanin-Villa, Juan David Núñez, David Rozo-Osorio, Isis Bonet, Mario Góngora, Mario A. Giraldo, Juan C. Tejada

**Affiliations:** a Artificial Intelligence and Robotics Research Group (IAR), Universidad EIA, Envigado, 055428, Colombia; b Department of Engineering Studies for Innovation, Universidad Iberoamericana Ciudad de México, Prolongación Paseo de la Reforma 880, Colonia Lomas de Santa Fé, Ciudad de México, 01219, Mexico; c Área de Industria, Materiales y Energía, Universidad EAFIT, Medellín, Colombia; d Institute for Artificial Intelligence (IAI), De Montfort University, Leicester, LE1-9BH, England, UK

**Keywords:** Soft robotics, Fin-Ray effect, Multi-robot systems, Cooperative manipulation, 3D-printed gripper

## Abstract

Soft robotic grippers are attractive for cooperative object transport in multi-robot systems because they tolerate positioning errors and reduce the risk of damage to fragile items. However, many Fin-Ray effect grippers lack integrated force feedback, require empirical tuning of geometry, and are not documented as open hardware, which limits their adoption in research and teaching platforms. This work presents the design, fabrication, instrumentation, and validation of an open-source Fin-Ray soft gripper tailored for caging-based manipulation with mobile robots. The gripper combines 3D-printed TPU fingers optimized via finite element analysis, a thin-film piezo-resistive force sensor, and an STM32-based proportional controller that regulates gripping force in real time. The complete hardware stack, including mechanical models, firmware, and a Python graphical interface for monitoring and control, is released as open design files. The sensor was characterized in the range from approximately 0.1 N to 5 N. A third-order polynomial calibration yields an average accuracy of 70.8 % over this interval, with reduced accuracy at very low forces, and an average coefficient of variation of 1.10 %, which indicates highly repeatable measurements. Static closed-loop tests against a rigid object show convergence to a 0.981 N force setpoint with small steady-state error. Dynamic interaction tests confirm that the controller compensates for external perturbations by adjusting the gripper aperture. Energy measurements reveal an average current consumption near 250 mA during regulation, with peaks around 1 A when rejecting disturbances. These results indicate that the proposed gripper is suitable as a low-cost, reproducible end-effector for cooperative manipulation experiments in multi-robot systems.

## Graphical abstract

**Figure:**
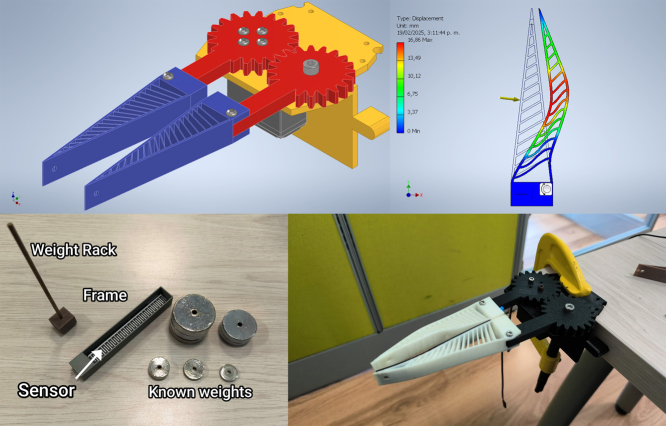

## Specifications table

**Table:** 

Hardware name	*Fin-Ray Soft Gripper*
Subject area	Educational tools and open source alternatives to existing infrastructure
Hardware type	Mechanical engineering and soft robotics
Closest commercial analog	HFDGXCI Mechanical Claw Fin-Ray gripper
Open source license	Creative Commons Attribution 4.0 International
Cost of hardware	$ 900 USD
Source file repository	https://doi.org/10.5281/zenodo.17917438

## 1 Hardware in context

Soft robotics has transformed modern manipulation by introducing compliance, adaptability, and safety into robotic interactions with uncertain environments [1], [2]. Unlike rigid manipulators that rely on precise kinematic models and high-bandwidth control, soft grippers exploit material deformation to conform to object geometries naturally. This capacity to grasp delicate, irregular, or fragile items makes them especially attractive for industrial, medical, and service applications where conventional rigid end-effectors are inadequate [3], [4].

Among soft gripper architectures, the Fin-Ray effect has emerged as one of the most effective and accessible principles for adaptive grasping. Adapted from the biomechanical structure of fish fins, the Fin-Ray fingers consist of two flexible sidewalls connected by transversal struts that redistribute applied forces and promote curvature toward the contact point [5], [6]. This simple geometry enables passive adaptation without complex control or sensing systems. Nevertheless, traditional Fin-Ray designs often lack integrated feedback, exhibit limited load capacity, and require empirical tuning of geometric parameters to balance compliance and stability [7], [8].

Recent research has addressed these limitations by embedding sensing capabilities and optimizing the structural topology of Fin-Ray grippers. Yang et al. [9] proposed a Fin-Ray finger with an integrated force sensor to monitor finger deformation and contact force. Liu and Adelson [10] introduced GelSight Fin-Ray, which incorporates a vision-based tactile sensor enabling high-resolution measurement of contact geometry and shear forces. Faris et al. [11] leveraged high-speed neuromorphic cameras and embedded fiducial patterns to achieve proprioceptive estimation of the finger’s internal state. Recently, Yao et al. [12] presented a generalized analytical model capable of performing sensorless prediction of both the deformed contour and the contact force of a Fin-Ray finger interacting with arbitrary object shapes. On the other hand, Cong et al. [13] developed a reconfigurable Fin-Ray architecture equipped with tactile skins and a four-bar actuation mechanism, enabling dexterous in-hand manipulation. Together, these advances demonstrate a rapid diversification of sensorized Fin-Ray architectures.

Such sensorized and reconfigurable architectures substantially improve grasp robustness, perception, and adaptability. However, they also increase fabrication complexity, material requirements, and overall system cost, factors that may constrain their adoption in open-source, low-budget, or educational robotic platforms.

In parallel, soft grippers have begun to play a fundamental role in multi-robot systems (MRS), where cooperative manipulation and transport are achieved through distributed coordination rather than single-arm grasping. These systems frequently employ the *caging* strategy, in which multiple robots enclose an object to constrain its motion while allowing compliant reorientation [14]. Effective caging requires each end-effector to maintain adaptive contact forces, avoid slippage, and tolerate positional uncertainties. However, conventional rigid or underactuated grippers lack the necessary compliance, and most soft designs are not optimized for mobile robotic platforms where compactness, low power consumption, and modularity are essential [15], [16], [17].

A previous work by Tejada et al. [18] proposed a cooperative manipulation framework that integrates Fin-Ray-inspired soft grippers into a leader–follower control scheme for object transport using two omnidirectional mobile robots. That work demonstrated the feasibility of caging-based control with Fin-Ray effect grippers. Building upon that foundation, the present research focuses on the physical realization and validation of an open-source Fin-Ray soft gripper optimized for cooperative object transport.

The proposed device combines 3D-printed flexible materials with embedded piezo-resistive force sensing and an STM32-based control platform. The contribution of this paper is to provide a low-cost, reproducible, two-finger gripper architecture specifically optimized for multi-robot caging applications. This design introduces an optimized 45° internal strut geometry that facilitates internal beam friction for increased structural rigidity under load, while allowing sufficient deformation to conform to the handled object. Additionally, the gripper is equipped with a flexible force sensor capable of delivering real-time force feedback for adaptive control. Finally, this work provides a documented open-hardware resource to the soft-robotics and multi-robot research community to enable reproducible studies in autonomous collaborative transport.

This paper is organized as follows: Section 2 presents the Design process of the Gripper, including its mechanical design, instrumentation, and software. Section 3 presents the files provided with the manuscript that allow the replication of the hardware. Section 4 presents the list of materials required to build the presented hardware. Section 5 presents the build instructions and the necessary information to assemble a replica of the described gripper. Section 6 presents how to operate the gripper with the provided code. Section 7 presents the validation of the gripper via static and dynamic tests, and the determination of the sensor’s accuracy and precision.

## 2 Hardware description

The proposed gripper was developed for object transport using the caging strategy in multi-robot systems, where objects are confined by multiple cooperative grippers rather than rigidly grasped. This approach increases robustness to positioning errors and uncertainty in object geometry. To support this interaction, the gripper integrates a compliant mechanical structure with embedded force sensing, enabling adaptive contact and force feedback during manipulation.

In contrast to conventional rigid grippers, the proposed design relies on mechanical compliance and force-based interaction instead of precision. The gripper is fully modular and fabricated using additive manufacturing and off-the-shelf components, making it significantly lower in cost and easier to reproduce than commercial or industrial alternatives.

Beyond its original application in multi-robot caging, the hardware serves as a flexible research platform that can be extended or modified for a wide range of manipulation and human–robot interaction studies.

• Low-cost, open-source platform for studying multi-robot caging and cooperative manipulation.
• Parametric and modular design enabling rapid customization and reuse.
• Suitable for research on force-based control and compliant interaction.
• Easily adaptable for educational, prototyping, and soft robotics applications.

### 2.1 Gripper design

The gripper is based on the Fin-Ray effect, a structure that enhances adaptivity and stability during grasping by conforming to various object geometries. This passive adaptation makes the gripper particularly effective for collaborative object transportation tasks. To replicate this behavior, the gripper finger consists of two main walls, each 100 mm in length, forming an isosceles triangle with an 83.3° apex angle, connected by parallel transversal struts.

**Fig. 1:**
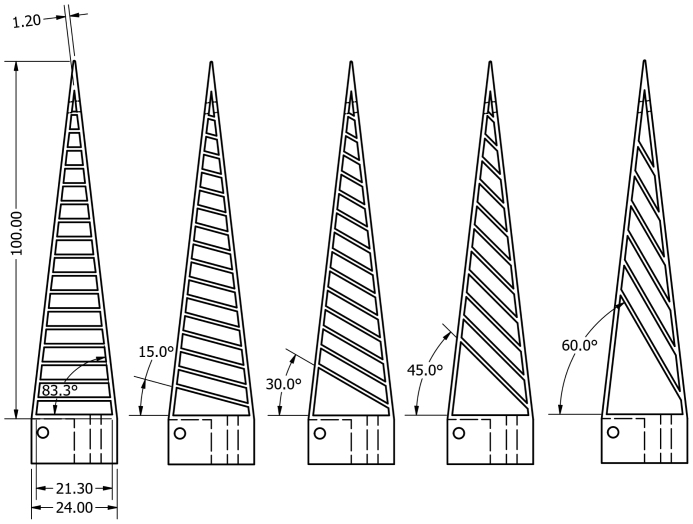
Fin-Ray effect grippers with transversal struts at different angles(0°, 15°, 30°, 45° and 60° respectively)

Since the orientation of these struts dictates the mechanical compliance of the structure, Finite Element Analysis (FEA) was conducted to evaluate strut angles ranging from 0° to 60° in 15° increments (see Fig. 1). Simulations were performed using TPU 95 A (Thermoplastic Polyurethane), a material selected for its elasticity and widespread use in FDM 3D printing. The material properties were sourced from MatWeb [19] and a characterization study by Haid et al. [20]. Each model utilized strut widths of 1 mm, 5 mm spacing, and a 3 N load applied over a 240 mm^2^ area located 52.5 mm from the base. The mesh included elements with a minimum size of 0.05 mm and a maximum rotation angle of 60°, with a grading factor of 1.5. The discretization captured the full 3D solid geometry to ensure an accurate representation of the deformation behavior under load.

The results, illustrated in Fig. 2, show both the displacement and the corresponding stress distributions for each configuration. The 45° strut configuration exhibited the highest compliance, with a maximum displacement of 22.26 mm. This configuration was selected as the optimization criterion because it maximizes the gripper’s ability to conform to handled objects while maintaining structural stability. The stress analysis confirmed that this angle keeps the material within its elastic limits during peak deformation, while the specific geometry facilitates internal beam friction under higher loads, providing the necessary rigidity to support heavier weights without sacrificing the soft nature of the gripper.

The fingers are manufactured using an Ender 3 V2 3D printer with 100% infill and generic TPU95 A filament. For printing TPU on the printer, direct-drive modifications are recommended due to the material’s elastic nature. This modification consists of moving the extrusion motor closer to the nozzle, effectively shortening the distance from the extruder to the hot end, making the process less prone to jamming or buckling on the way to the nozzle, and making filament retractions more precise since the filament would stretch less.Fig. 2Fin-Ray effect gripper with transversal struts at different angles Finite Elements Displacement results. **(a)** 0° angle. **(b)** 15° angle. **(c)** 30° angle. **(d)** 45° angle. **(e)** 60° angle.Fig. 2

**(a):**
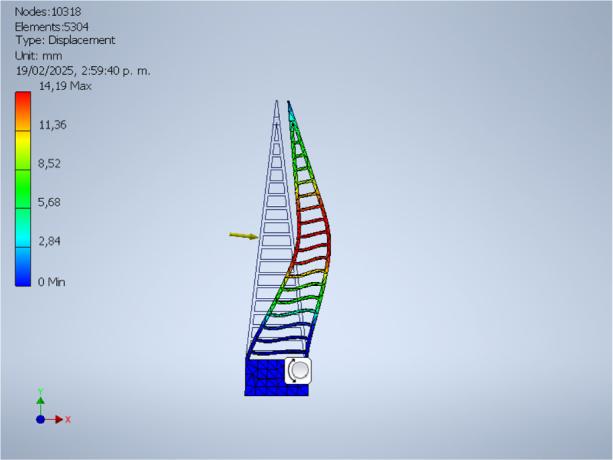
.

**(b):**
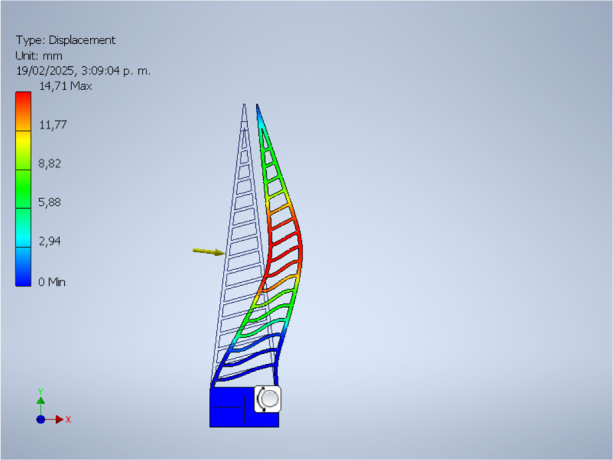
.

**(c):**
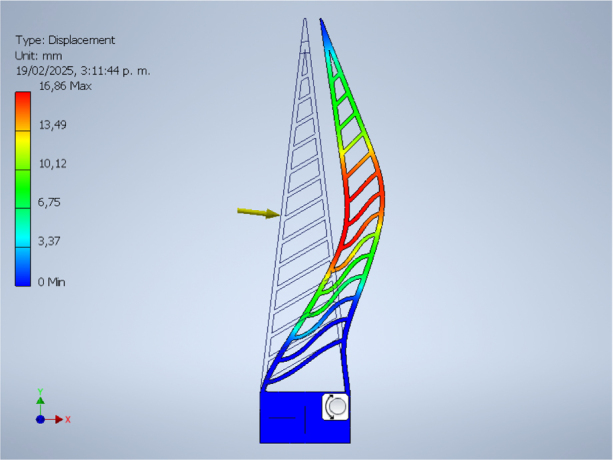
.

**(d):**
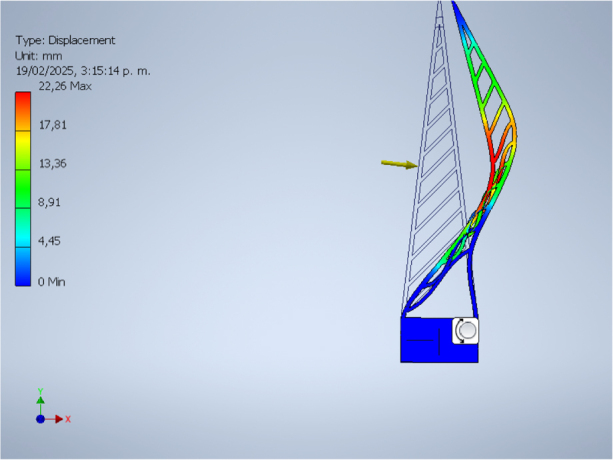
.

**(e):**
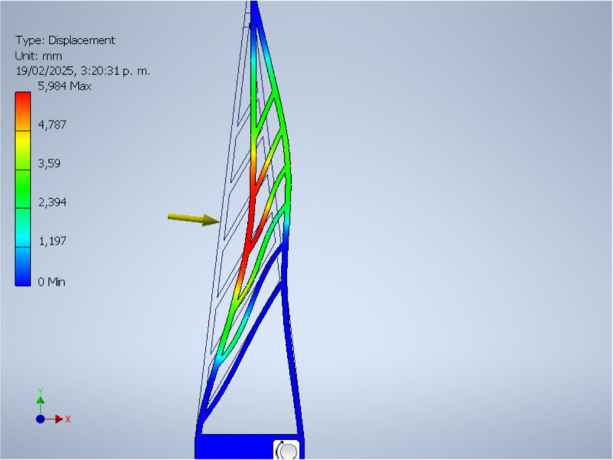
.

Additionally, the following set of printing parameters was specified in CuraSlicer to generate the corresponding G-code for the 3D printer:

1. **Layer height:** 0.2 mm
2. **Printing temperature:** 220 °C
3. **Nozzle diameter:** 0.4 mm
4. **Printing speed:** 20 mm s^−1^
5. **Bed temperature:** 30 °C
6. **Cooling:** Disabled
7. **Retraction:** Disabled
8. **Combing mode:** All

For integration with the YAHBOOM ROSMASTER X3 mobile robot platform, a custom 3D-printed PLA mounting bracket was designed. The system uses an HK15138 servomotor with a torque rating of 4.3 kg*cm at 6 V to actuate the gripper via two geared arms in a 1:1 transmission, each with 20 teeth, a 20° pressure angle, and a 2.25 mm module, ensuring symmetric finger motion. This allows for object centering when actuating the gripper, with an intended operating range of cubic objects from approximately 20×20×20cm up to 30×30×30cm. Although the gripper was initially designed for 20cm boxes used in caging tasks, the geometry and compliance of the Fin-Ray structure allow for stable grasping of moderately larger objects within this range. The selected system layout of two fingers driven by a single servo was chosen for its simplicity; the final assembly of the system can be seen in Fig. 3. The printing files for the system are available in the supplementary material for this article.

Fig. 3Assembly of gripper mechanism. **(a)** CAD. **(b)** 3D printed gripper.Fig. 3

**(a):**
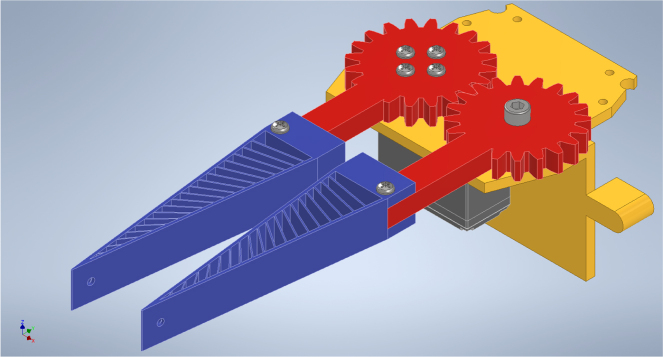
.

**(b):**
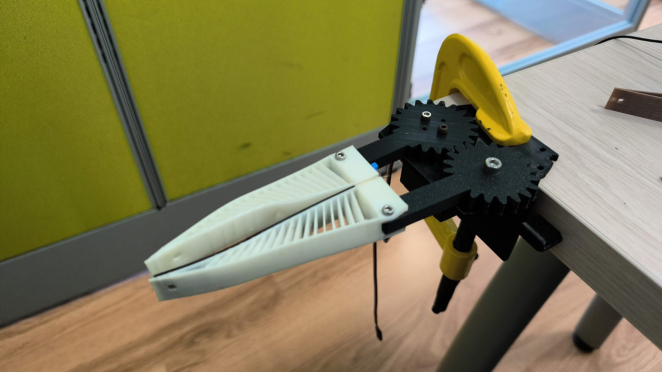
.

### 2.2 Instrumentation

The gripper was instrumented using a similar approach to that of Yang et al. [9], with a piezoresistive thin-film force sensor. The instrumentation is essential to be able to gather information on the contact force the gripper has with the object being transported, information necessary to determine if the gripper is actually gripping the object or not, and to correct the angle of the servomotor accordingly. The sensor is branded as ***H52 Pressure Sensor*** by JESSINIE and has a rectangular form factor of 110 mm × 15 mm. The maximum measurement limits are rated at 20–10 000 g.

**Fig. 4:**
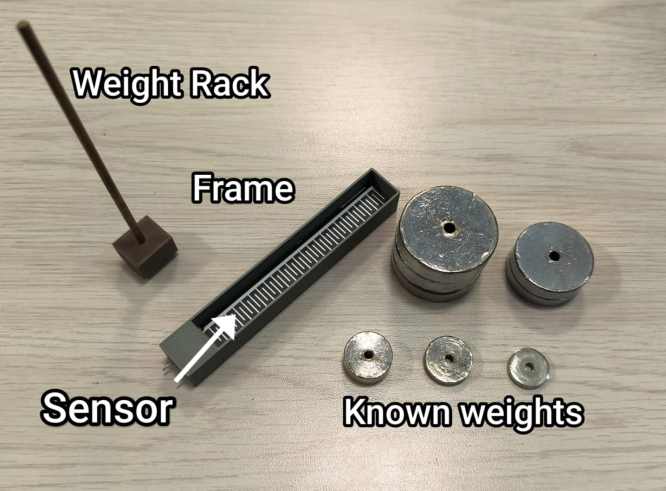
Calibration fixture.

**Fig. 5:**
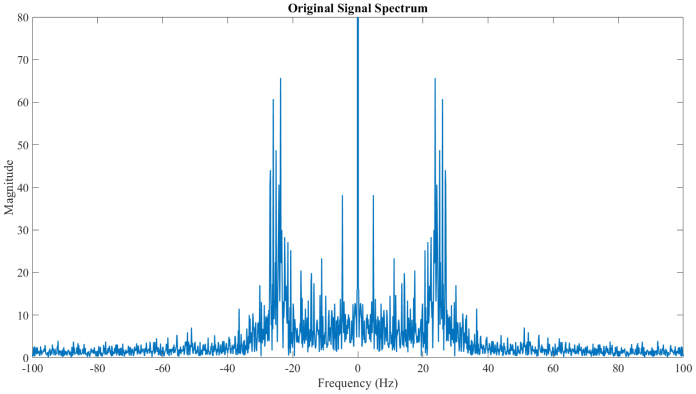
Fast Fourier Transform of the piezo-resistive sensor while idle.

The following stages were performed to characterize the piezoresistive sensor. These steps preserve the original experimental flow and can be replicated for similar thin-film devices. Due to the high variability observed during early tests, mainly caused by uneven contact pressure, a dedicated calibration fixture was designed (Fig. 4) to ensure the applied load was focused on the active (striped) sensing region (see Fig. 5).

1. Fixture Assembly and Sensor Placement
  The sensor was mounted inside the rigid calibration frame, ensuring that the active region aligned precisely with the loading point. The weight rack allowed the application of known vertical loads in a controlled and repeatable manner.
2. Electrical Configuration and Baseline Measurement
  The sensor was wired to a voltage-divider circuit. Its output was monitored with an oscilloscope to verify correct operation and quantify baseline noise. Idle measurements showed approximately 0.1 V of noise, around 4.5 % of the usable range (1.1 V–3.3 V).
  To identify dominant noise frequencies, a Fourier Transform was computed:
3. Filtering Stage and Signal Conditioning
  The FFT revealed noise concentrated around 20 Hz–30 Hz, motivating the design of a 20 Hz FIR low-pass filter. A 200-tap moving-average filter was implemented on an STM32F407G-DISC1 board. With the ADC sampling at 4000 Hz, the effective cutoff matched the target 20 Hz.
  The filtered output exhibited significantly reduced fluctuations:
4. Incremental Load Application and Data Acquisition
  Loads were applied in 50 g increments up to 500 g. For each load, the output voltage was sampled and averaged. These averaged values produced the calibration dataset used for curve fitting. The calibration was carried up to 500 g because the servomotor used in the gripper cannot generate gripping forces approaching the upper range of the sensor; therefore, forces beyond this value would not occur during regular operation.
5. Calibration Curve Fitting
  The recorded voltage-weight pairs were fitted using a third-order polynomial model. This polynomial should be interpreted as a data-driven empirical calibration curve rather than a physics-based constitutive model of the piezoresistive film. The measured response presented a dual-curvature trend over the calibrated interval, especially between the low-force region and the medium-force region. Therefore, a first- or second-order regression was not sufficient to reproduce the observed nonlinear behavior. Conversely, higher-order polynomials were avoided because they could overfit the limited calibration dataset and introduce unstable extrapolation outside the measured range. The third-order model was consequently selected as a practical compromise between flexibility and robustness, and it was implemented in firmware only within the experimentally calibrated range. The resulting curve (Fig. 7) was then implemented in firmware to estimate gripping force during operation.
  The fitted relation is given by Eq. (1).
  (1) $$W=\frac{9.81\left(-2092.8V^{3}+18651V^{2}-55684V+55852\right)}{1000}$$

**Fig. 6:**
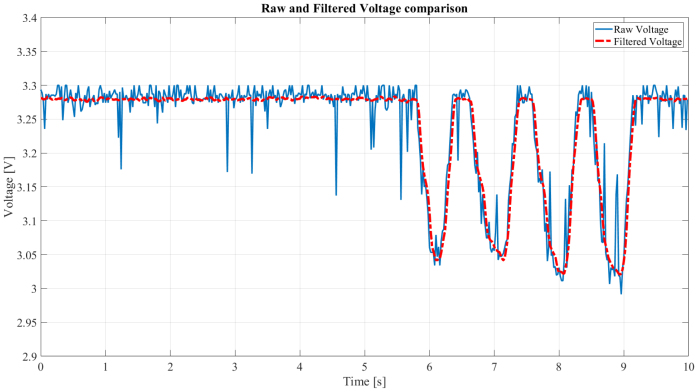
Comparison between raw and filtered voltage measurements.

To achieve the force feedback with the selected servomotor, a TB6612FNG motor driver breakout board was used. This driver was powered by 6 V (the servomotor’s nominal voltage) and allowed the PWM signal from the STM32 development board to reach the motor safely. The TB6612FNG was selected because it provides a dual H-bridge topology with low output on-resistance, sufficient current capability for the servomotor’s transient loads, and reliable level shifting between the microcontroller’s logic domain and the motor supply. Unlike simpler transistor-based stages or less efficient drivers, the TB6612FNG ensures stable PWM transmission, reduced electrical noise, and protection against back-EMF, making it suitable for precise force-controlled actuation. The full connection diagram can be seen in Fig. 8 (see Fig. 6).

**Fig. 7:**
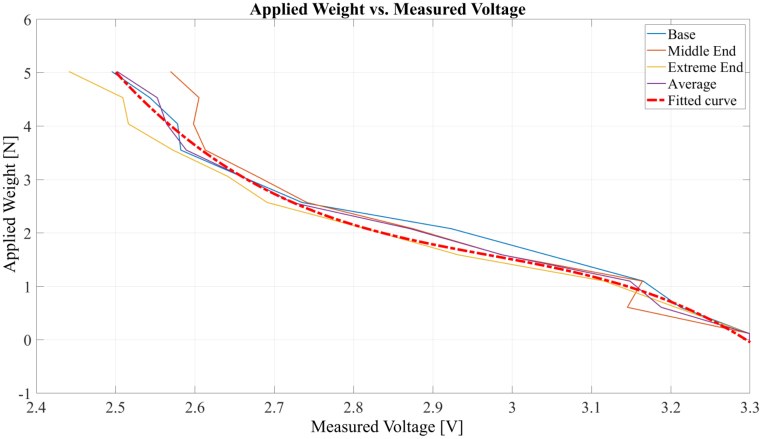
Calibration curve for the piezo-resistive sensor.

**Fig. 8:**
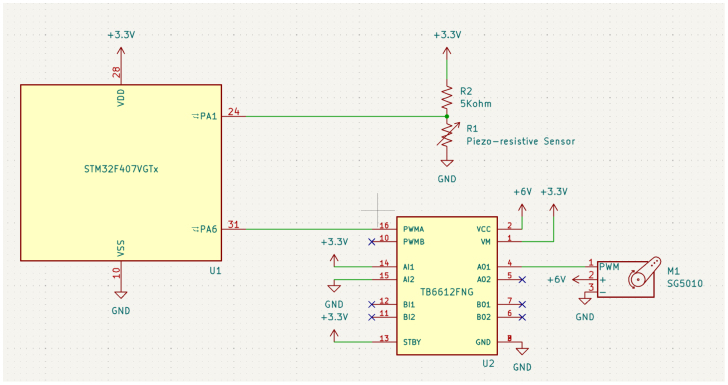
Electronic Diagram for Gripper controller.

### 2.3 Software and control system

Firmware was developed in C using STM32CubeIDE. The control system operates as a finite-state machine with four states: *START*, *CONTROL*, *IDLE*, and *END*. Mode selection is achieved via USB commands (’s’, ‘i’, ‘c’).

During the *CONTROL* state, a proportional controller adjusts the PWM duty cycle according to the error between the desired setpoint and the measured force. Safety limits are enforced to prevent servo overdrive. Real-time telemetry, comprising sensed force, setpoint, error, and control action, is streamed via USB for visualization.

The PWM frequency for the servomotor was set to 50 Hz, which is the standard control frequency for hobby servomotors. According to the servomotor datasheet, the neutral position corresponds to a pulse width of 1500 µs. A variation of 500 µs from this neutral value produces an angular displacement of approximately ±45°. Therefore, the minimum and maximum command limits were set to 500 µs and 2500 µs, respectively, corresponding to the 180° nominal actuation range of the servomotor.

A proportional control scheme was implemented for this stage. The force measurement obtained from the sensor is compared against the desired setpoint to compute the instantaneous error. This error is then multiplied by an empirically tuned proportional gain, $K_{p}=0.1$, and the resulting output is used to update the servo’s pulse-width command. Finally, the computed pulse-width is constrained to remain within the predefined operational limits of the actuator.

**Fig. 9:**
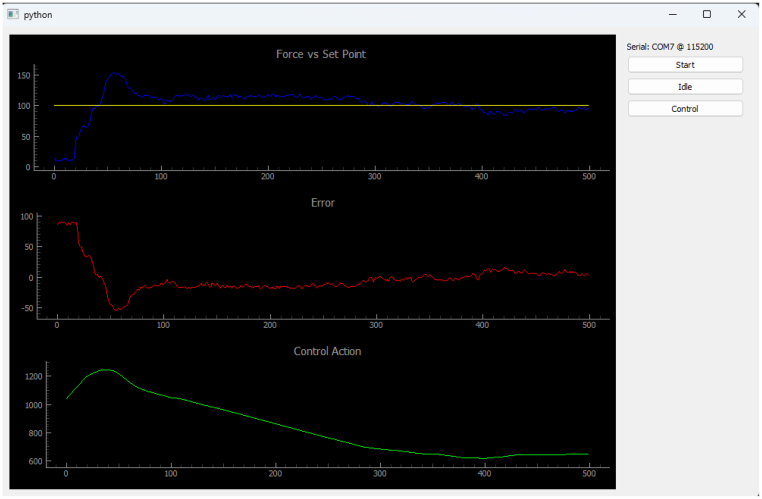
Python graphic user interface.

On the computer side, a Python graphical user interface was developed to control the gripper states by sending serial commands to the STM32 board (Fig. 9). The interface also displays the measured force, setpoint, control error, and PWM pulse width in real time. The program was developed using the PyQtGraph library to provide a reliable and straightforward front end. When the interface is closed, the most recent plotted data are saved automatically as a CSV file for later analysis.

## 3 Design files summary

**bracket.stp**: Parametric CAD model of the structural bracket used to mount and align the gripper assembly with the actuation system, designed for easy modification and reuse (see Table 1).

**bracket.stl**: Mesh file generated from the bracket CAD model, optimized for additive manufacturing using standard FDM 3D printers.

**Table 1:** Design files summary.

Design filename	Version	File type	Open source license	Location of the file
bracket.stp	v1	CAD file	Creative Commons Attribution 4.0 International.	Available with the article.
bracket.stl	v1	Print file	Creative Commons Attribution 4.0 International.	Available with the article.
GearedArm_Driven.stp	v1	CAD file	Creative Commons Attribution 4.0 International.	Available with the article.
GearedArm_Driven.stl	v1	Print file	Creative Commons Attribution 4.0 International.	Available with the article.
GearedArm_Driver.stp	v1	CAD file	Creative Commons Attribution 4.0 International.	Available with the article.
GearedArm_Driver.stl	v1	Print file	Creative Commons Attribution 4.0 International.	Available with the article.
Gripper_Finger.stp	v1	CAD file	Creative Commons Attribution 4.0 International.	Available with the article.
Gripper_Finger.stl	v1	Print file	Creative Commons Attribution 4.0 International.	Available with the article.
Assembly.mp4	v1	Video file	Creative Commons Attribution 4.0 International.	Available with the article.
assembly_thumbnail.png	v1	Figure	Creative Commons Attribution 4.0 International.	Available with the article.
plotter.py	v1	Python script	Creative Commons Attribution 4.0 International.	Available with the article.
STM32_firmware.zip	v1	STM32 Cube IDE project	Creative Commons Attribution 4.0 International.	Available with the article.
README.md	v1	Detailed Instructions	Creative Commons Attribution 4.0 International.	Available with the article.

**GearedArm_Driven.stp**: CAD model of the driven geared arm, which transmits motion from the actuation mechanism to the gripper fingers.

**GearedArm_Driven.stl**: Printable version of the driven geared arm, suitable for direct fabrication without further processing.

**GearedArm_Driver.stp**: CAD model of the driver geared arm, designed to interface with the motor shaft and generate controlled mechanical motion.

**GearedArm_Driver.stl**: STL file of the driver geared arm prepared for 3D printing and mechanical integration.

**Gripper_Finger.stp**: Parametric CAD model of the compliant gripper finger, incorporating the geometric features required for adaptive grasping.

**Gripper_Finger.stl**: Mesh file of the gripper finger optimized for additive manufacturing and experimental prototyping.

**Assembly.mp4**: Video demonstrating the complete mechanical assembly process of the gripper, including the order of components and alignment steps.

**assembly_thumbnail.png**: Representative image extracted from the assembly video, used as a visual reference for the assembled gripper.

**plotter.py**: Python script used to process experimental data and generate plots related to the gripper’s performance and sensor response.

**STM32_firmware.zip**: Complete STM32CubeIDE project containing the firmware for sensor acquisition, control implementation, and actuator driving.

**README.md**: Documentation file providing detailed instructions for fabrication, assembly, firmware deployment, and system operation.

## 4 Bill of materials summary

See Table 2.

**Table 2:** Bill of materials summary.

Designator	Component	Number	Cost per unit (USD)	Total cost (USD)	Source of materials	Material type
BOM-01	HK15138 servomotor	1	4.00	4.00	TowerPro	Composite
BOM-02	STM32F407G-DISC1 development board	1	20.00	20.00	STMicroelectronics	Semiconductor
BOM-03	TB6612FNG motor driver breakout board	1	14.00	14.00	Sparkfun	Semiconductor
BOM-04	Force sensor (H52)	1	12.00	12.00	JESSINIE	Composite
BOM-05	Tesa 4965 double-sided tape	1	16.00	16.00	Tesa	Polymer
BOM-06	THT 5 kΩ resistor	1	0.05	0.05	Generic	Ceramic
BOM-07	Mounting bracket (3D printed)	1	0.50	0.50	Custom 3D print	Polymer
BOM-08	Gripper fingers (3D printed)	2	0.25	0.50	Custom 3D print	Polymer
BOM-09	Geared arms (3D printed)	2	0.25	0.50	Custom 3D print	Polymer
BOM-10	Round servomotor bracket	1	1.50	1.50	Honbay	Metal
BOM-11	MR105ZZ bearing	1	0.70	0.70	Generic	Metal
BOM-12	M3 × 10 screws	4	0.125	0.50	Generic	Metal
BOM-13	M3 × 15 screws	2	0.25	0.50	Generic	Metal
BOM-14	M5 × 8 screws	4	0.125	0.50	Generic	Metal
BOM-15	M5 × 20 screw	1	0.10	0.10	Generic	Metal
BOM-16	M5 hex nut	1	0.10	0.10	Generic	Metal
BOM-17	M3 hex nuts	2	0.05	0.10	Generic	Metal
BOM-18	ROSMASTER X3 mobile robot (optional)	1	799.00	799.00	Yahboom	Composite
BOM-19	Regulated DC power supply, 6 V, ≥2 A (optional)	1	15.00	15.00	Generic	Non-specific

## 5 Build instructions

The gripper assembly comprises 3D-printed parts and off-the-shelf components. Editable CAD models, STL files, and firmware are provided as supplementary material. Table 2 lists the main components and materials required for reproduction, while Table 3 summarizes the 3D-printed parts and their associated design files.

STL and STEP versions of each 3D-printed part are provided in the public repository to facilitate reproduction and modification of the proposed gripper.

**Assembly steps:**

**Table 3:** Summary of 3D-printed parts and associated design files.

Part	File name	Material	Recommended print orientation
Gripper finger	Gripper_Finger.stl/.stp	TPU 95A	Finger lying on its lateral side, with the flat outer wall on the build plate and the transversal struts facing upwards.
Mounting bracket	Bracket.stl/.stp	PLA	Base of the bracket on the build plate, with the servo cavity facing upwards.
Geared arm (driver)	GearedArm_driver.stl/.stp	PLA	Gear face on the build plate to ensure accurate tooth geometry.
Geared arm (driven)	GearedArm_driven.stl/.stp	PLA	Gear face on the build plate; the bore for the MR105 bearing should remain vertical.

1. **3D printing.** Print the bracket and geared arms in PLA using the orientations listed in Table 3. Print the two gripper fingers in TPU 95 A with 100% infill, as described in Section 2. After printing, remove any support material and verify that the gear teeth are free of defects.
2. **Servo installation.** Mount the HK15138 servomotor onto the PLA bracket using the M5 × 8 screws. The servo output shaft should be oriented towards the front of the bracket. Attach the round servomotor bracket to the servo shaft following the manufacturer’s instructions.
3. **Driver geared arm.** Fix the driver geared arm to the servo bracket using the appropriate M3 screws. The arm should be aligned such that its neutral position corresponds approximately to the gripper’s symmetric open configuration. Screws should be tightened until the joint is snug and free of play, avoiding excessive torque that could crack the PLA part.
4. **Driven geared arm and bearing.** Press-fit the MR105ZZ bearing into the bore of the driven geared arm. Then mount this arm onto the bracket using the M5 × 20 screw and M5 hex nut, ensuring that the bearing allows free rotation. Verify that the two gears mesh correctly without binding and that both arms rotate symmetrically.
5. **Finger attachment.** Attach one TPU finger to each geared arm using M3 screws and M3 hex nuts. The fingers should be oriented such that their inner concave surfaces face each other and are aligned when the gears are in the neutral position.
6. **Sensor placement.** Mount the piezo-resistive force sensor on the inner surface of one finger using Tesa 4965 double-sided tape. The active (striped) area of the sensor should be centered in the expected contact region with the object. It is recommended to apply gentle, uniform pressure during adhesion to avoid air gaps.
7. **Electrical wiring.** Connect the force sensor to the voltage-divider circuit on the STM32F407G-DISC1 board, and wire the TB6612FNG driver between the STM32 PWM output and the servomotor, following the schematic in Fig. 8. The servo is powered from the regulated 6 V supply, while the STM32 board is powered from USB or a separate regulated 5 V supply, with all grounds tied together.
8. **Firmware deployment and test.** Flash the firmware described in Section 2 onto the STM32 board using STM32CubeIDE. With the gripper unloaded, power the system and verify communication via USB. Using the Python GUI, command the system into *START* and *CONTROL* modes, and check that the fingers open and close smoothly and that the force readings change when manually applying a load to the sensor.

### Safety considerations.

The gripper is intended for benchtop laboratory use at low forces, but the following safety aspects should be considered:

• **Mechanical safety.** The moving fingers can create pinch points between the geared teeth. Users should keep their hands, hair, and loose clothing away from the closing region during operation.
• **Electrical safety.** The servomotor should be powered from a regulated 6 V DC supply with appropriate current limiting (typically ≥2 A). All connections must be checked for correct polarity and insulation before powering the system to avoid short circuits or component damage.
• **Thermal considerations.** Prolonged operation at high load or with the gripper stalled against a rigid object can cause the servomotor to heat up. It is recommended to operate within the calibrated force range and to allow the servo to cool between extended tests.

These guidelines are sufficient to safely assemble and operate the gripper in typical research and educational environments.

## 6 Operation instructions

To ensure proper operation, the object to be grasped should be placed approximately at the center of the gripper aperture, with its faces aligned to the inner surfaces of the fingers. This positioning allows the Fin-Ray structure to deform symmetrically and maximizes contact with the sensing surface.

No additional calibration is required once the device is assembled; the force sensor is calibrated prior to integration, and its calibration curve remains valid throughout normal operation.

During use, the force setpoint must remain within the calibrated and mechanically feasible range of the system (0–3 N). However, based on the sensor characterization, forces below approximately 1.5 N should be interpreted mainly for contact detection and trend monitoring, rather than for accurate absolute force regulation. For experiments requiring quantitative force feedback, setpoints above this threshold are recommended. If a setpoint corresponding to forces near or above 500 g is commanded, the gripper will be unable to reach the target. In such cases, the control law saturates, and the servomotor may remain stalled, which can lead to unnecessary heating and reduced actuator lifespan. It is therefore recommended to avoid setpoints beyond the operational region.

To operate the gripper:

1. Power the STM32 board and connect it to a PC via USB.
2. Launch the Python control and visualization tool.
3. Select the operating mode (’s’ = start, ‘i’ = idle, ‘c’ = control).
4. Set the desired force reference within the safe range.
5. Place the object between the fingers and allow the controller to regulate the gripping force.
6. Monitor the real-time force and control signals displayed in the GUI.

Safety note: Avoid placing hands or loose items near the gears, and do not operate the gripper under prolonged stall conditions, as the servomotor may heat up. The device is intended for benchtop experimental use under low-force conditions.

## 7 Validation and characterization

### 7.1 Accuracy and precision

To evaluate the quality of the calibrated sensor, its output was compared against the accurately applied weights. The analysis focused on two key performance indicators: *accuracy* and *precision*. Measurements were collected in increments of 50 g, obtaining ten readings per increment. For each weight interval, the accuracy was computed using the absolute error, percentage error, and accuracy expressions shown in Eqs. (2)–(4).

(2) $$E_{abs}=\left|W_{\mathrm{measured}}-W_{\mathrm{true}}\right|$$

(3) $$E_{\text{\%}}=\left(\frac{E_{\mathrm{abs}}}{W_{\mathrm{true}}}\right)\times 100$$

(4) $$A=max\left(0,100-E_{\text{\%}}\right)$$

Table 4 summarizes the accuracy results for each weight interval starting with the weight rack’s weight. The sensor exhibits low accuracy in the lower end of the tested range; however, accuracy improves progressively as the applied weight increases. The overall mean accuracy obtained throughout the full range was 70.812 %. The reduced accuracy below approximately 1.5 N can be attributed to the combined effect of the nonlinear response of the piezoresistive film, the low signal-to-noise ratio at small loads, and the mechanical interaction between the flexible TPU finger and the sensor surface. At low applied forces, small variations in local contact area, pressure distribution, and sensor placement produce voltage changes that are comparable to the baseline noise of the measurement circuit. Although the calibration fixture was designed to focus the applied load on the sensor’s active region, it cannot fully reproduce the distributed, deformable contact conditions that occur when the sensor is mounted on the compliant Fin-Ray finger. This explains why the absolute force estimate is less reliable at the lower end of the calibrated range, while accuracy improves as the applied force increases and the sensor response becomes more distinguishable from the noise floor.

For the intended multi-robot caging application, the sensor is not used as a metrological instrument for high-accuracy absolute force measurement. Instead, its main function is to detect contact, identify force trends, and provide repeatable feedback for adjusting the gripper aperture during cooperative object confinement. In caging-based manipulation, the object is constrained by the geometry and compliance of multiple grippers, so the control strategy places greater emphasis on stable contact, repeatability, and relative changes in force than on the exact absolute force value. Therefore, force readings below approximately 1.5 N should be interpreted mainly as contact and trend information, whereas quantitative force regulation is more reliable above this threshold. The low coefficient of variation reported in the precision analysis supports this interpretation, since it indicates that the sensor response is repeatable even when its absolute accuracy is limited at low forces.

**Table 4:** Accuracy results for each weight interval.

Real mass [g]	Real weight [N]	Absolute error [N]	Percentage error [%]	Accuracy [%]	Standard deviation (σ) [N]
12.060	0.118	0.165	139.498	−39.498	0.000
62.060	0.609	0.656	107.676	−7.676	0.000
112.060	1.099	0.341	30.980	69.020	0.033
162.060	1.590	0.198	12.436	87.564	0.016
212.060	2.080	0.098	4.687	95.313	0.019
262.060	2.570	0.139	5.406	94.594	0.027
312.060	3.061	0.238	7.768	92.232	0.023
362.060	3.552	0.194	5.475	94.525	0.032
412.060	4.042	0.142	3.503	96.497	0.024
462.060	4.533	0.048	1.050	98.950	0.053
512.060	5.023	0.130	2.592	97.408	0.048

Precision was assessed using the coefficient of variation (CV), which relates the standard deviation and the mean of the ten measurements per interval, as defined in Eq. (5).

(5) $$CV=\left(\frac{\sigma }{\mu }\right)\times 100$$

Table 5 shows the calculated precision for each weight. The CV values remain relatively small across the tested range, indicating that the sensor produces consistent measurements around the mean. The average precision was 1.0996 %, demonstrating that the sensor exhibits stable behavior with minimal variation among repeated measurements.

Overall, the precision values fall within the range [0.82 %, 4.32 %], confirming that the sensor maintains a consistent response for repeated measurements. Despite its limited accuracy in the low-force region, the device demonstrates high precision, making it dependable for applications where repeatability is more critical than absolute accuracy.

**Table 5:** Precision results calculated using the coefficient of variation.

Weight [N]	Precision [%]
0.118	0.000
0.609	0.000
1.099	4.323
1.590	1.129
2.080	0.977
2.570	1.1035
3.061	0.821
3.552	0.945
4.042	0.621
4.533	1.188
5.023	0.987

### 7.2 Force-feedback controller validation

To evaluate the performance of the implemented PID force-feedback controller, two experimental scenarios were considered. First, static tests were conducted to assess whether the gripper could reach and maintain a desired contact force under stationary conditions. Second, dynamic interaction tests were performed to evaluate the controller’s ability to reject external disturbances and recover the force setpoint after perturbations.

#### 7.2.1 Static tests

To evaluate the performance of the force-feedback controller under stationary conditions, the gripper was tested against a rigid object. The objective of this test was to verify whether the PID controller could reliably drive the gripper to a desired force setpoint and maintain it during sustained contact.

A target force of 0.981 N, equivalent to the weight of a 100 g mass, was selected. This value was chosen as a representative setpoint for a caging manipulation strategy, where the gripper maintains a stable contact force to entrap objects during multi-robot transport rather than supporting the full object weight through friction.

The selection of this setpoint also ensures design coherence between the mechanical and electronic subsystems. The HK15138 servomotor, with a torque of 4.3 kg⋅cm, provides sufficient mechanical advantage to reach the sensor’s maximum calibrated range of approximately 5 N. Selecting 0.981 N allows the system to operate within a linear and reliable region of the low-cost sensor while remaining well below the motor’s thermal and torque limits during sustained contact with the target object.

**Fig. 10:**
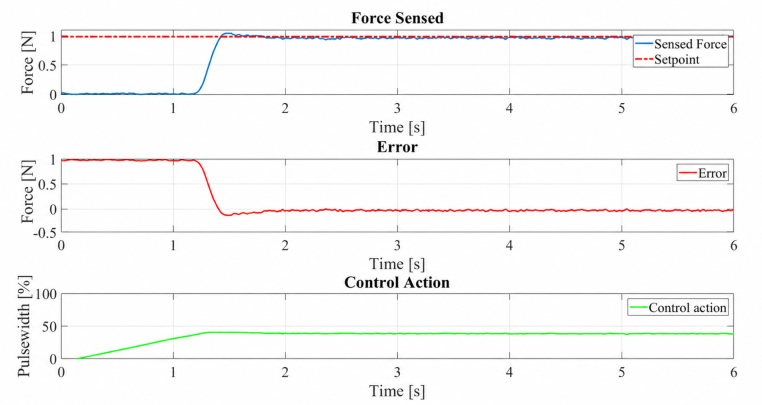
Static test of the PID force-feedback controller showing the sensed force, force error, and control action during convergence to the 0.981 N setpoint.

During the test, the servomotor actuated the mechanism until the measured force approached the predefined setpoint. Once contact with the object was established, the PID controller continuously adjusted the gripper position to stabilize the contact force around the reference value. The resulting force response over time is shown in Fig. 10, where the system successfully converged to the desired setpoint.

The system exhibits an initial contact delay of approximately 1.12 s, corresponding to the time required for the gripper to close and establish contact with the object. This delay is mainly associated with the initial gripper–object separation and may vary depending on object size and geometry. After contact, the PID controller rapidly increases the sensed force toward the desired setpoint of 0.981 N. The force response reaches a maximum value of approximately 1.01 N to 1.03 N, corresponding to a reduced overshoot of approximately 3 % to 5 %. The response then converges to the reference value with a settling time of approximately 1.08 s, measured from the instant of contact. During steady-state operation, the force remains close to the reference, with residual fluctuations mainly associated with sensor noise, mechanical compliance, friction, and the finite resolution of the servomotor.

The error response confirms this behavior. Before contact, the error remains close to the reference force because no interaction force is measured. Once contact occurs, the error rapidly decreases toward zero and exhibits only a small negative peak associated with the transient overshoot. After the settling period, the error remains close to zero, indicating that the integral action of the PID controller effectively reduces the steady-state deviation. Similarly, the control action initially increases as the servomotor closes the gripper and then stabilizes around an operating pulse width of approximately 40 % to 45 %, with small corrective variations produced by the PID controller. These results show that the implemented PID force-feedback controller achieves stable convergence, reduced overshoot, low steady-state error, and a suitable control effort for the intended caging-based manipulation task.

#### 7.2.2 Dynamic interaction

Dynamic tests were performed to evaluate the behavior of the PID force-feedback controller when external disturbances modified the contact force applied to the gripper. Unlike the static test, where the gripper interacted with a rigid object under stationary conditions, this experiment assessed the controller’s ability to reject perturbations and recover the desired force setpoint after sudden changes in the interaction force.

The procedure consisted of first allowing the gripper to reach the desired force setpoint of approximately 1.0 N. Once stable contact was achieved, external disturbances were manually applied to the gripper. These perturbations were introduced to emulate unexpected changes in the contact condition, such as those that may occur during cooperative object transport when the manipulated object shifts, rotates, or experiences uneven load distribution. Larger disturbances increased the measured contact force, requiring the controller to open the gripper and reduce the applied load. Conversely, reductions in contact force required the controller to close the gripper further to recover the desired interaction force.

**Fig. 11:**
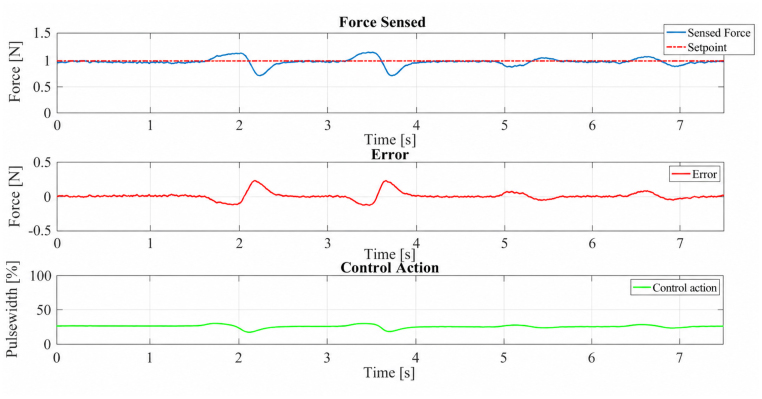
Dynamic interaction test of the PID force-feedback controller showing the sensed force, force error, and control action during external perturbations.

Due to limitations in the available experimental equipment, the exact magnitude of the manually applied external disturbances could not be independently measured. However, the embedded force sensor provided quantitative information about the internal force variation during the test. As shown in Fig. 11, the applied perturbations produced two main force excursions approximately between 0.68 N to 1.24 N, followed by smaller perturbations within an approximate range of 0.86 N to 1.12 N. Despite these changes, the PID controller was able to drive the sensed force back toward the 1.0 N setpoint after each disturbance.

The first major disturbance occurs after the system has reached stable contact, producing a transient increase in the sensed force followed by a reduction below the reference value. In response, the control action modifies the servomotor pulse width to compensate for the force deviation and restore the desired contact condition. A similar behavior is observed during the second major disturbance, where the force again deviates from the reference but returns to the setpoint after the corrective action of the controller. For these larger perturbations, the recovery time was approximately 0.75 s, measured as the time required for the sensed force to return close to the reference after the disturbance peak.

The error signal confirms the disturbance-rejection capability of the controller. During stable operation, the error remains close to zero and within an approximate 5 % band around the desired force. When external perturbations are applied, the error exhibits positive and negative peaks depending on whether the sensed force falls below or rises above the setpoint. After each perturbation, the error decreases again toward zero, indicating that the integral action contributes to reducing persistent deviations, while the derivative action improves the transient response during abrupt force variations.

The control-action signal also provides evidence of the PID controller’s behavior. During the major disturbances, the pulse width increases or decreases according to the sign and magnitude of the force error, allowing the gripper to open or close as required. After each correction, the control signal returns to a stable operating region, with smaller variations associated with residual sensor noise, mechanical compliance, and contact uncertainty. These results show that the implemented PID force-feedback controller is capable of maintaining stable contact forces under dynamic interaction conditions. Therefore, the proposed control strategy is suitable for caging-based manipulation, where the gripper must preserve adaptive contact with the object rather than exert a large constant gripping force.

### 7.3 Energy consumption

To complete the validation of the gripper’s behavior, the energy consumption of the servomotor was analyzed. Because the gripper often attempts to reach an angle that cannot be achieved due to an object obstructing motion, it was expected that the motor would draw currents above its nominal operating value.

Experimental measurements confirmed this behavior. During static force-control operation, the motor consumed an average current of approximately 250 mA, with peaks reaching up to 1000 mA when compensating for load variations. On the other hand, the current consumption during idle and initial positioning was close to zero, indicating that the primary energy demand occurs only while actively maintaining or adjusting the gripping force.

## 8 Conclusions and future work

This work presented the design, fabrication, instrumentation, and validation of an open-source Fin-Ray soft gripper tailored for cooperative manipulation tasks in multi-robot systems. The gripper combines a FEA-tested Fin-Ray geometry with embedded piezoresistive force sensing and an STM32-based proportional controller, enabling real-time force feedback while maintaining low cost, accessibility, and ease of replication.

The mechanical evaluation demonstrated that a 45°transverse strut configuration maximizes structural compliance, thereby improving adaptation to irregular object geometries. The calibrated sensing system showed high measurement precision (average CV of 1.10 %), although accuracy at low force levels remains limited due to the piezo-resistive film’s nonlinear behavior. Static validation confirmed that the controller reliably converges to the desired gripping forces, while dynamic tests demonstrated effective disturbance rejection, enabling the gripper to adapt its opening angle to external force variations. Energy consumption measurements indicated that most power usage occurs during active regulation, with peaks reaching up to 1 A during high-load conditions.

The results confirm that the proposed gripper meets the requirements for adaptive object caging in cooperative mobile robotic platforms: it is compliant, low-cost, responsive to contact forces, and reproducible without specialized equipment.

Future work will address various limitations identified during development. First, a custom PCB will be designed to replace the development board and integrate the sensor interface, power management, and motor driver into a compact embedded system. Second, although the gripper is functional, it remains easily back-drivable, which can lead to unintentional motion, inaccurate angle estimation, and increased servo stress; therefore, future iterations will explore non-back-drivable transmissions or small reduction mechanisms. Finally, the manufacturability of the soft fingers will be expanded beyond FDM 3D printing by investigating resin printing, silicone casting, and injection molding techniques to improve material consistency, durability, and mechanical repeatability as key factors for multi-robot deployments.

## CRediT authorship contribution statement

**Santiago Velasquez:** Validation, Software, Project administration, Methodology, Investigation, Conceptualization. **Alejandro Toro-Ossaba:** Validation, Methodology, Investigation, Formal analysis, Conceptualization. **Daniel Sanin-Villa:** Writing – original draft, Visualization, Investigation, Funding acquisition, Conceptualization. **Juan David Núñez:** Writing – original draft, Validation, Software. **David Rozo-Osorio:** Writing – review & editing, Software, Resources. **Isis Bonet:** Writing – original draft, Validation, Methodology, Investigation. **Mario Góngora:** Writing – review & editing, Writing – original draft, Project administration, Formal analysis. **Mario A. Giraldo:** Writing – original draft, Validation, Conceptualization. **Juan C. Tejada:** Resources, Methodology, Formal analysis.

## Ethics statements

The authors declare that this work complies with the journal ethics policies and did not involve human subjects or animal experiments.

## Funding

This work was supported by Universidad EIA (grant INVIM0082024), Universidad Iberoamericana Ciudad de Mexico, INIAT and SECIHTI (grant 1228748).

## Declaration of competing interest

The authors declare that they have no known competing financial interests or personal relationships that could have appeared to influence the work reported in this paper.
